# A methodological note on the CYP-guides randomized controlled trial for the treatment of major depression

**DOI:** 10.3389/fpsyt.2022.888065

**Published:** 2022-10-11

**Authors:** Georgios Sideridis, Ghadah Alkhadim

**Affiliations:** ^1^Boston Children's Hospital, Harvard Medical School, Boston, MA, United States; ^2^Department of Primary Education, National and Kapodistrian University of Athens, Athens, Greece; ^3^Department of Psychology, College of Arts, Taif University, Riyadh, Saudi Arabia

**Keywords:** CYP-GUIDES database, clinical trial, major depression, length of stay, admission rate

## Abstract

Genetically guided therapy for major depression has been recently recommended but has resulted in null effects. We hypothesized that a potential confounding variable for not finding differences in Length of Stay and Re-admission rate in a major depression clinical trial is the equivalence between treatment protocols in the standard treatment (S) and *CYP2D6* genotype (G) treatment groups. The two treatment protocols (i.e., type and degree of substrate drugs) were contrasted using a latent class analysis (LCA) model. Specifically, an LCA model specifying the presence of two classes, namely, the G and S groups was estimated with the intercepts of the 30 prescribed drugs freely estimated. This model was compared to a constrained latent class model in which the two treatment protocols (intercept terms) were contrasted to be equivalent between the two treatment groups. Results indicated that there were no significant differences between G and S treatment groups in the types and number of drugs administered. Consequently, the lack of finding significant differences in length of stay and readmission rate may likely be attributed to the equivalence of the treatment protocols.

## Introduction

Major depression (MDD) is one of the most common disorders in the United States. Amongst adults aged 18 or older 21 million have repored at least one majore depressive episode with this number mounting to 8.4% of the total adult population in the U.S. (1). The respective numbers for adolescents (aged 12–17) was 2.9 million, representing 12% of the total adolescent population. Interestingly, 34% of the adults diagnosed with depression and 58% of the adolescents failed to receive any form of treatment for their major depressive episode (2). The picture is further complicated as the phenomenon is more prevalent in females compared to males. The consequences of depression on wellbeing and functioning have been well-documented with links to disability and physical health problems, poor quality of life, substance abuse, homelessness, and even suicide [e.g., (3)]. Consequently it is imperative that treatments are consistently developed and tested to alleviate the negative effects of depression for both the individual and society.

Recent proposals for the treatment of major depression suggested the need to adjust medication based on information provided by *Cytochrome Psychotropic Genotyping* (CPG). Based on the CYP-Guides controlled RCT registered in *ClinicalTrials.gov* using identified NCT 02120729 (4), a standard treatment protocol was contrasted to a *CYP2D6* genotype treatment for patients with major depressive disorder (MDD). Primary and secondary outcomes were the length of stay (LOS) and re-admission rate (RAR) for which statistical evidence provided support for null models. The authors suggested that “confounders may have obscured the effects of pharmacogenetic guidance” and freely provided the data as a means for exploring the presence of confounding variables toward elucidating the observed null findings. In their design, they first randomized 1,500 patients with major depression using a 2:1 ratio onto a “genetically-guided” therapy group (termed G) and a standard care group (termed S), with the assignment to group performed after genotyping. Inclusionary criteria were age>18, a diagnosis of major depression, and the ability to understand the project's requirements and execute the trial's protocol. They classified the drugs administered using colors so that the standard treatment group would receive the typically prescribed drugs (colored green) and the patients with *CYP2D6* functional status would receive major substrate drugs (in red color) or minor substrate drugs (in yellow color) as well as some degree of standard drugs depending on *CYP2D6* functional status. The authors transmitted to physicians information and guidance on drug selection per patient using the Electronic Medical Record (EMR) for a total of 30 drugs. Among them, 16 had a major CYP2D6 substrate dependence, seven a minor one, and seven a non-substrate dependence. We hypothesized that a potential confounding variable in the present RCT may reside in the quality and quantity of the psychotropic medications administered in the two groups, thus, we tested the hypothesis of null differences across psychotropic medication protocols using a confirmatory latent class model.

The presenst study is important for several reasons. First, the original clinical trial was published as an open source dataset in Mendeley with the goal: “to assess the impact of clinical decision support on utilization of psychiatric resources for treatment of severe depression requiring hospitalization.” Our paper attempts to challenge the utility of the CYP-guides clinical trial by providing a concinving argument as to why the original study failed to identify differences between standard therapy and genetically-guided therapy groups. Consequently, the present study elaborates on the presence of the originally presented null effects and suggests that future clinical trials re-examine the roles of genetically-guided therapy as the latter was confounded heavily in the Ruano et al. study. Thus, understanding the likely cause of the null findings will inform future research on the topic, rather than disergarding genetically-guided treatment as ineffective.

## Methods

Data on the dependent variables RAR and LOS as well as on medication protocols were drawn from the CYP-GUIDES database [see (5)]. The data are available in the data availability section.

### Data analysis

Data were analyzed using a Latent Class Analysis (LCA) approach using known groups (i.e., treatment protocols G and S) as the goal was to compare and contrast treatment protocols in level. An alternative would be the classic Analysis of Variance test (ANOVA) but it would treat the medications independent of each other and not within the latent variable framework of all medications belonging to the same latent factor. Two latent class models were specified, (a) a freely estimated one suggesting group non-equivalence across levels in the prescribed medications, and, (b) a constrained model in which intercept terms across medications in the two groups were forced to be equivalent (i.e., same intercept terms). Evidence of the superiority of a model was provided using loglikelihood difference tests (distributed as a chi-square statistic) and several information criteria as specified in the work of Masyn (6). Amongst information criteria, the Akaike Information Criterion (AIC), the Bayesian Information Criterion (BIC), the Consistent AIC (CAIC), the Approximate Weight of Evidence (AWE) criterion, and the Schwartz Information Criterion (SIC) all favor a model when values are smaller compared to a competing model; the cmP(k) index is 1 (or close to one) for the preferred model and 0 for a non-preferred model; a large estimate on the Bayes Factor (BF) points to the preferred model. All analyses were conducted using Mplus 8.8 (7). Our decision to include both inferential (loglikelihood difference test) and information criteria was guided by the fac that both methodologies have pros and cons and may result in differential conclusions (8).

## Results

### Equivalence between G and S using information criteria and omnibus statistical test

As mentioned above, the freely estimated LCA model and the constrained LCA model (specifying treatment equivalence) were contrasted using both information criteria and inferential statistics to answer the research question “Are there differences in the administration of medication treatment protocols?”. Results unequivocally favored a conclusion of no significant differences between the two treatment protocols, challenging the premises of the designed methodologically speaking protocols. Using the information criteria, the constrained model was the preferred model by being associated with better fit and for also being more parsimonious [AIC_Constrained_ = 22,750.00, AIC_Free_ = 22,790.00; BIC_Constrained_ = 22,883.04, BIC_Free_ = 23,051.79; CAIC_Constrained_ = 22,914.04, CAIC_Free_ = 2,3112.79; AWE_Constrained_ = 23,171.08, AWE_Free_ = 23,618.57; BF_Constrained_ > 15, BF_Free_ < 0.001; cmPk_Constrained_ = 1.00, cmPk_Free_ = 0.00]. The cmPk showed a preference for the constrained model (equivalent intercepts) and so did the Bayes factor. Thus, collectively, the estimation of additional intercept terms was not justified, and based on the principle of parsimony (9), the constrained model suggesting an equivalence between intercept terms across G and S groups was the preferred choice with these data.

**Figure 1:**
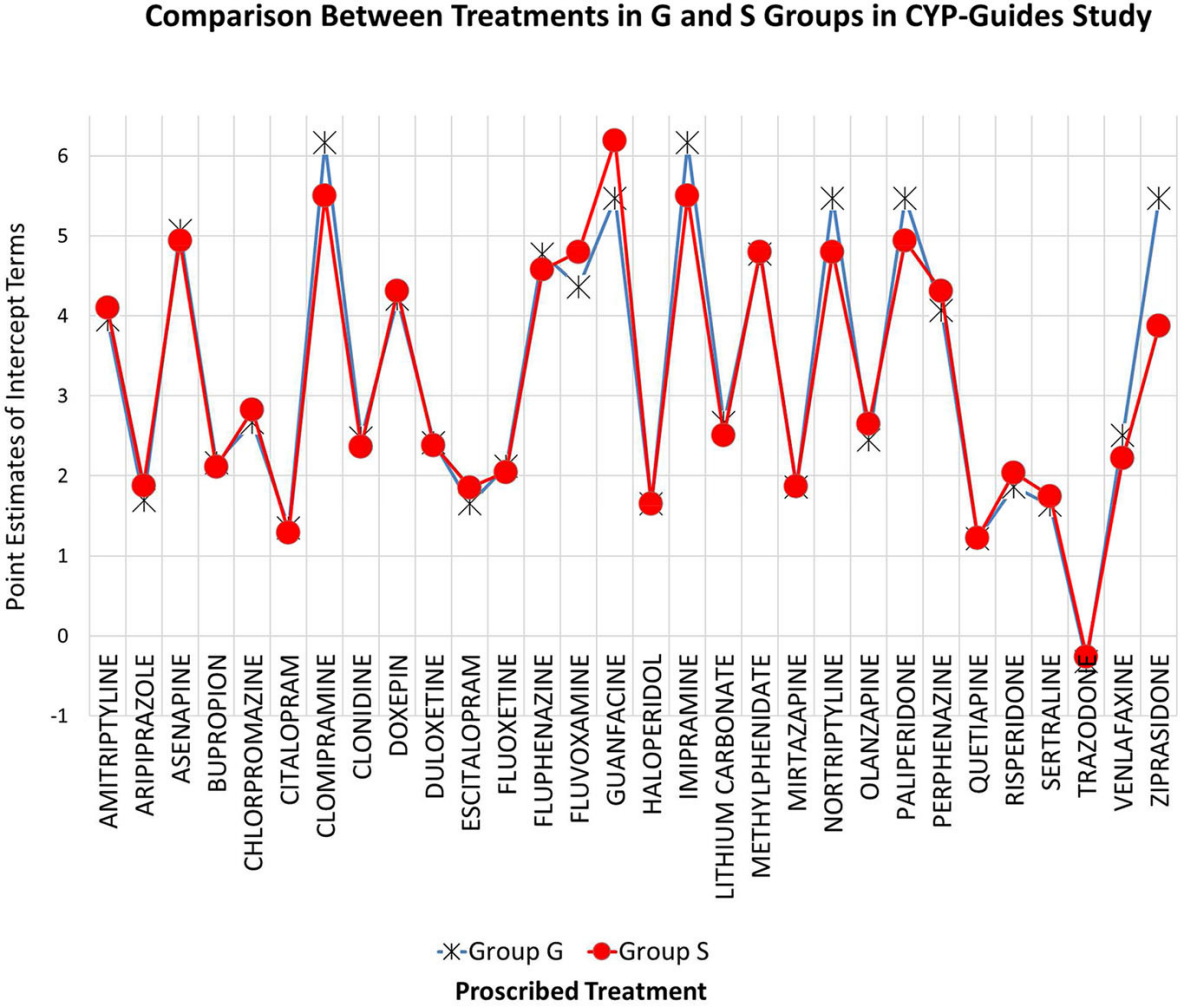
Known groups latent class model for evaluating the equivalence of treatment medications across G and S groups in CYP-guides study. Estimates reflect levels in the administration of drugs across treatment groups.

This conclusion was further challenged using inferential statistics. Superior model fit was judged using a loglikelihood difference test. Results indicated that there were no significant differences between G and S treatment groups in the administration of various treatments [Chi-square (30) = 20.00, *p* = 0.917]. Figure 1 shows the intercept terms of the two classes, the standard treatment one (S) and the genotype one (G). As shown in the figure, minuscule differences were observed across groups on their intercept terms, most likely reflecting random variations around those estimates. This finding is certainly robust considering the excessive levels of power that are likely operative with a sample size of 1,500 participants, enhancing our confidence in the conclusion of null differences across G and S treatment groups.

## Conclusions

The authors of the trial pointed to the need to investigate the presence of confounding variables in the CYPD RCT trial as a means of understanding the lack of significant differences between the G and S treatment groups in the treatment of major depression. Results indicated that the prescription of drugs that were initially thought to be specialized in the two groups as a function of their genotype was no different from the standard treatment groups. Thus, in the presence of equivalent medical treatments and random assignment, the lack of finding significant differences in the two outcome variables is likely expected. Further confounding factors for the presence of null effects on the outcome variables may relate to the performance of pharmacogenetic testing (10) as threats to their internal validity have been recently raised [see also (11)]. Although this limitation could be addressed by testing the metabolic performances of the CYP isoenzymes, this information was neither present in the original study nor to the present authors. We believe, however, that this potential confounding would likely be accounted for by random assignment although we could not be certain of that fact in the absence of relevant evidence knowing that even random assignment can fail. Another potential confounding variable relates to the presence of concurrent medications that may inhibit 2D6 that, likely exerts salient effects on depression. Future studies could examine the need to model physician characteristics as a random variable or explore treatment implementation to further investigate the presence of confounding variables that challenge the conclusion of null effects due to treatment. Regardless, the present study suggests that the null effects observed when contrasting standard therapy vs. genetically-guided therapy are unjustified and rooted to the equivalence of the medical protocols administered. Thus, future studies need to further explore the roles of genetically guided therapy and pharmacogenetic testing that has shown promise (11).

## Data availability statement

Data are part of a national database and have been posted in Mendeley (https://data.mendeley.com/datasets/25yjwbphn4/1).

## Ethics statement

Ethical approval was not provided for this study on human participants because data are part of a national database and have been posted in Mendeley (https://data.mendeley.com/datasets/25yjwbphn4/1). Approval of the study is within their published works describing the data. Written informed consent for participation was not required for this study in accordance with the national legislation and the institutional requirements.

## Author contributions

GS conceptualized the study and contributed to data analyses and the write-up of the manuscript. GA contributed to data analyses and the write-up of the quantitative sections. All authors approved the final draft of the manuscript.

## Funding

This project was funded by Taif University Researchers Supporting Project number (TURSP-2020/334), Taif University, Taif, Saudi Arabia. This work was conducted as part of the requirements for the Foundations of Clinical Research Program at Harvard Medical School by GS.

## Conflict of interest

The authors declare that the research was conducted in the absence of any commercial or financial relationships that could be construed as a potential conflict of interest.

## Publisher's note

All claims expressed in this article are solely those of the authors and do not necessarily represent those of their affiliated organizations, or those of the publisher, the editors and the reviewers. Any product that may be evaluated in this article, or claim that may be made by its manufacturer, is not guaranteed or endorsed by the publisher.
